# The Great Imitator: Latent Neurosyphilis Revealed After Initiation of the Immunosuppressive Drug Secukinumab

**DOI:** 10.7759/cureus.18462

**Published:** 2021-10-03

**Authors:** Allison Duncan, Nicholas Zingas, Anas Ahmed, Roger Shih

**Affiliations:** 1 F. Edward Hébert School of Medicine, Uniformed Services University of the Health Sciences, Bethesda, USA; 2 Internal Medicine Residency Program, Wright State University, Dayton, USA; 3 Department of Internal Medicine/Infectious Disease, Wright-Patterson Medical Center, Wright-Patterson AFB, USA

**Keywords:** palm and sole rash, secukinumab, immunosuppression, latent syphilis, neurosyphilis, syphilis

## Abstract

Syphilis is a multi-organ system bacterial infection caused by the bacterium *Treponema pallidum*. Syphilis can advance through four clinical stages: primary, secondary, latent, and tertiary. Once in the tertiary stage, mortality is seen in up to 58% of individuals. Here, we present a case of latent neurosyphilis manifesting after initiation of the immunosuppressive medication secukinumab, a monoclonal antibody that antagonizes interleukin-17A.

A 66-year-old male with type II diabetes mellitus, hyperlipidemia, and rheumatoid arthritis presented to the emergency department for a right lower quadrant abdominal cellulitis at the site of his insulin pump. On examination, a non-blanching papular rash on the palms and soles with several scaling papules was discovered. No visible pustules, oral lesions, or perirectal lesions were seen. Neurological examination was noncontributory. His past medical history revealed initiation of secukinumab for the management of rheumatoid arthritis two months prior to presentation. The rash developed six weeks after starting secukinumab. Basic laboratory tests, including a complete blood count, thyroid panel, renal function panel, fasting blood glucose, electrolytes, and C-reactive protein, were within normal limits. A hepatic panel revealed mildly elevated alkaline phosphatase, alanine transaminase, and erythrocyte sedimentation rate Westergren level. Laboratory tests for hepatitis B, hepatitis C, HIV-1, *Chlamydia trachomatis*, and *Neisseria gonorrhoeae *all returned negative. A rapid plasma reagin (RPR) titer returned positive at 1:128, and a serum *Treponema pallidum* Ab returned reactive. Lumbar puncture serologies demonstrated a positive Venereal Disease Research Laboratory (VDRL) test. The patient was diagnosed with latent neurosyphilis and started on intravenous crystalline penicillin G for three weeks.

A thorough history, comprehensive physical examination, and basic workup should be performed in any individual prior to immunosuppressive medication initiation. On initial presentation, our patient had an isolated rash on the palms and soles, which is classical for secondary syphilis. The specific manifestations seen in syphilis depend upon the timing, site, and immune status of the individual. Due to its ability to have a variety of presentations, syphilis should always remain on the differential for any physician caring for immunocompromised individuals. Again, initiation of immunosuppressive medications, such as the monoclonal antibody secukinumab, can result in the reactivation of previously dormant infections. As physicians, we must carefully screen our patients prior to initiating immunosuppressive agents to prevent disease reactivation.

## Introduction

Syphilis is a multi-organ system infection caused by the bacterium *Treponema pallidum* [1,2]. Syphilis can advance through four clinical stages, primary, secondary, latent, and tertiary, each with unique presenting symptoms and treatments [3]. The prognosis of syphilis significantly improved with the discovery of penicillin in 1928; however, the United States has seen a slow increase in the prevalence of syphilis since 2001 [4,5]. We present a case of latent neurosyphilis discovered after the initiation of secukinumab, a monoclonal antibody that antagonizes interleukin-17A. This article was previously presented as a meeting abstract and poster presentation at the DAGMEC 22nd Annual Virginia C Wood Resident Research Forum at Wright State University Boonshoft School of Medicine in Dayton, OH, USA, in May 2021.

## Case presentation

A 66-year-old male with rheumatoid arthritis, type II diabetes mellitus, and hyperlipidemia presented to the emergency department for a right lower quadrant abdominal cellulitis at the site of his insulin pump. On examination, a 3 x 1.5 cm non-tender erythematous region with nodularity was appreciated on his right lower abdomen. Further examination revealed a non-blanching papular rash on bilateral palms and soles with several scaling papules. No visible pustules and oral or perirectal lesions were noted. Neurological examination was noncontributory. His past medical history revealed initiation of the medication secukinumab for the management of rheumatoid arthritis two months prior to presentation. According to the patient, the palm and sole rashes developed six weeks after starting secukinumab. Further history revealed no sexual activity in the previous year but a distant history of *Chlamydia trachomatis* infection in the 1990s. Per the patient, the infection was successfully treated, and he tested negative for HIV at that time. Basic laboratory tests, including a complete blood count, thyroid panel, renal function panel, fasting blood glucose, electrolytes, and C-reactive protein, were within normal limits. A hepatic panel revealed mildly elevated alkaline phosphatase, alanine transaminase, and erythrocyte sedimentation rate Westergren level. Laboratory tests for hepatitis B, hepatitis C, HIV-1, *Chlamydia trachomatis*, and *Neisseria gonorrhoeae* all returned negative. The patient was started on oral doxycycline for his cellulitis with infectious disease follow-up in one week. At the follow-up appointment, a rapid plasma reagin (RPR) titer returned positive at 1:128, and a serum *Treponema pallidum* Ab returned reactive. Lumbar puncture was then performed, and serologic examination of cerebrospinal fluid demonstrated a positive Venereal Disease Research Laboratory (VDRL) test. The patient was diagnosed with latent neurosyphilis and started on intravenous crystalline penicillin G for three weeks duration. At follow-up after completion of penicillin treatment, the patient's rash had resolved, and repeat neurological examination was benign.

## Discussion

The discovery of penicillin in 1928 radically improved the prognosis of syphilis in the 20th century [4]. However, since 2001, the prevalence of syphilis has continued to slowly rise in the United States. In 2018, the country saw the highest number of reported cases since the previous century, with a total of 115,045 cases nationwide. This was a 13.3% increase from the previous year [5]. 

**Figure 1:**
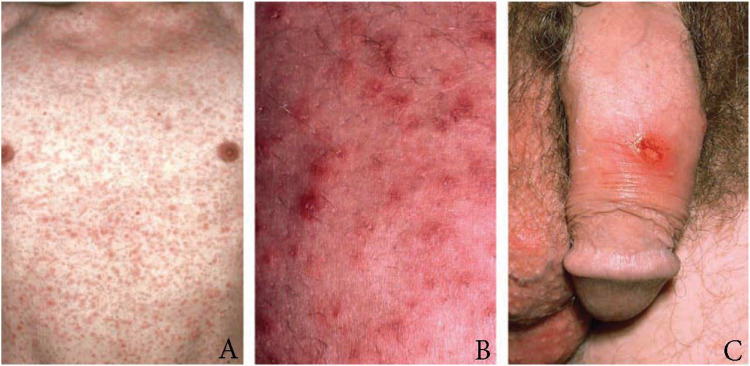
Physical symptoms of early (primary and secondary) syphilis. A: Maculopapular rash. B: High-power view of maculopapular rash as seen in secondary syphilis. C: Painless penile chancre as seen in primary syphilis. Nyatsanza F, Tipple C. Clinical Pictures of Patients with Early Syphilis. Clinical Photographs of Patients with Early Syphilis: a) Typical Maculopapular Rash on the Chest; b) Skin with Secondary Syphilis Rash at High Magnification; c) Penile Chancre. Syphilis: Presentations in General Medicine. *Clinical Medicine.* 2016, 16:184. doi: https://doi.org/10.7861/clinmedicine.16-2-184

Syphilis, known to early modern medical providers as “the great imitator,” is a sexually transmitted bacterial infection that may present with a number of various symptoms and timing of presentation after infection [6]. When left untreated, syphilis will progress through four clinical stages, primary, secondary, latent, and tertiary, each having their own unique presenting symptoms and treatments. The clinical symptoms of early (primary and secondary) syphilis can be visualized in Figures 1A-1C. If the central nervous system is infected, neurosyphilis develops. This may occur at any of the four clinical stages; however, it is more common to occur in the latter stages as the infection progresses when left untreated.

Immune system impairment causes an increased risk of further or quicker disease progression [7-9]. Thus, patients positive for HIV, patients taking immunosuppressive medications, and patients with immunopathology are more likely to present with the classic symptoms of secondary or tertiary syphilis.

## Conclusions

This case highlights the importance of thoroughly screening immunocompromised patients for syphilis, especially if the patient has a history of unprotected sexual intercourse and/or sexually transmitted infection(s). In this case, initiation of the immunosuppressive medication secukinumab intended to treat the patient’s rheumatoid arthritis resulted in the reactivation of latent syphilis. While the presentation of a papular palm and sole rash appeared to be secondary syphilis, further testing, which resulted in positive RPR, reactive serum *Treponema pallidum* Ab, and positive VDRL, revealed neurosyphilis.

This further stresses the importance of always screening for and including syphilis on the differential for any suspicious signs or symptoms in an individual on immunosuppressive therapy, due to its ability to have a variety of manifestations depending on the timing, immune status, and sites involved.

## References

[1] Kent ME, Romanelli F (2008). Reexamining syphilis: an update on epidemiology, clinical manifestations, and management. Ann Pharmacother.

[2] Peeling RW, Mabey D, Kamb ML, Chen XS, Radolf JD, Benzaken AS (2017). Syphilis. Nat Rev Dis Primers.

[3] Toptan T, Ozdilek B, Kenangil G, Ulker M, Domac FM (2015). Neurosyphilis: a case report. North Clin Istanb.

[4] Gaynes R (2017). The discovery of penicillin—new insights after more than 75 years of clinical use. Emerg Infect Dis.

[5] (2021). Sexually transmitted disease surveillance 2018. https://www.cdc.gov/std/stats18/syphilis.htm.

[6] Barnett R (2018). Syphilis. Lancet.

[7] Proudfoot M, McLean B (2013). Old adversaries, modern mistakes: neurosyphilis. Pract Neurol.

[8] Lee SJ, Park HK, Kim YS (2019). A case of rapid progressive neurosyphilis in patient with ankylosing spondylitis who is treating anti-interleukin 17A monoclonal antibody, secukinumab. J Rheum Dis.

[9] Uslu U, Heppt F, Sticherling M (2017). Secondary syphilis infection under treatment with ustekinumab. Clin Exp Dermatol.

